# Blood Circulating miRNAs as Cancer Biomarkers for Diagnosis and Surgical Treatment Response

**DOI:** 10.3389/fgene.2019.00169

**Published:** 2019-03-11

**Authors:** Samantha Filipów, Łukasz Łaczmański

**Affiliations:** Ludwik Hirszfeld Institute of Immunology and Experimental Therapy, Polish Academy of Sciences, Wrocław, Poland

**Keywords:** miRNA, biomarker, cancer, diagnosis, treatment response

## Abstract

miRNAs can function as potential oncogenes or tumor suppressors. Altered expression of these molecules was correlated with the occurrence of many cancer diseases and therefore they are considered a molecular tool for non-invasive cancer diagnosis and prognosis. We searched for analyses concerning expression of blood circulating miRNA in cancer patients. The studies comprised of at least two miRNA expression measurements: before and after the surgical therapy were considered. We summarized latest reports on evaluation of the efficiency of anticancer therapy through observation of changes in expression of miRNA circulating in blood of patients treated with surgery alone. Twenty one research studies were identified. Thirty one different miRNAs were pointed out as potential both diagnostic and treatment response biomarkers since their deregulated expression before therapy returned to normal after receiving the treatment. Published data revealed a potential of circulating miRNA to become a tool giving a clinical follow up information on the efficiency of applied therapy. However, more observational studies on post-operative circulating miRNA expression changes are necessary.

## Introduction

### miRNA Biogenesis

**FIGURE 1:**
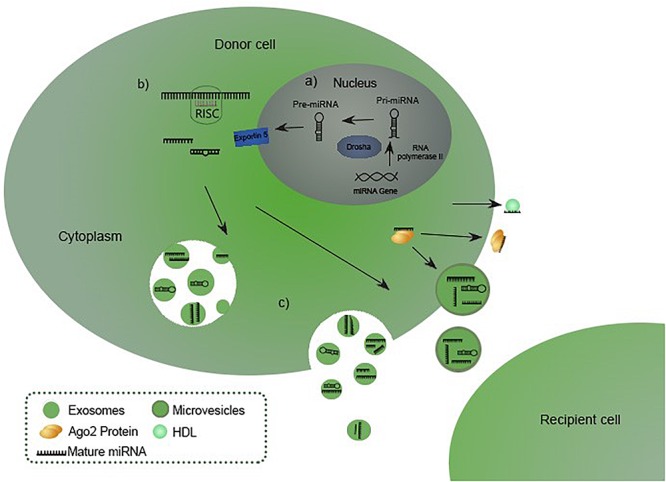
miRNA biogenesis, maturation and releasing (Sohel, 2016). **(A)** miRNA biogenesis starts in the nucleus with transcription of a pri-miRNA which is subsequently processed by Drosha into precursor miRNA (pre-miRNA), exported to the cytoplasm in pre-miRNA-Exp-5-RAN-GTP complex. **(B)** Pre-miRNA is processed into a mature miRNA by Dicer RNase III endonuclease. Eventually the molecule hybridizes with a RISC complex to fulfill its biological functions. **(C)** Matured miRNA is released in the extracellular environment incorporated into the vesicles or bound to Ago or HDL (van Rooij and Kauppinen, 2014; Lan et al., 2015; Sohel, 2016).

miRNAs are members of a large class of non-coding RNA originally discovered in *Caenorhabditis elegans* in 1993, initially regarded as ncRNA (non-coding RNA). Molecules received their current name after proving their existence in other eukaryotic organisms in 2001 (Leva et al., 2014), although the occurrence of RNA molecules working as translation inhibitors in eukaryotic cells was firstly mentioned by Heywood et al. (1974) and Lages et al. (2012). Since then, miRNAs presence has been demonstrated in virtually all species (Leva et al., 2014). miRNA coding genes are widespread in genome, except for chromosome Y. Up to half of them are located in clusters on chromosomes with a common promoter. Neighboring genes are cotranscribed, which results in forming miRNA families (Lan et al., 2015). Promoting regions of genes are predicted *in silico* and are transcribed by RNA II polymerase as primary 33 nucleotides-long transcripts called pri-miRNA. In the canonical pathway for miRNA biogenesis, primary miRNAs (pri-miRNAs) are processed by RNase III Drosha and RNA-binding protein DGCR8 into 70 nucleotides long precursor miRNAs (pre-miRNAs). Pre-miRNAs are transferred to the cytoplasm by exportin-5 (Exp-5), which binds RAN-GTP protein and nucleoporins in the nucleus allowing the transport. The eventual maturation is performed by Dicer RNase III, endonuclease that cleaves the pre-miRNAs into mature single-stranded miRNAs. Molecules are integrated into the RNA induced silencing complex (RISC) to fulfill their biological functions (van Rooij and Kauppinen, 2014; Lan et al., 2015). A schematic representation of the canonical miRNA biogenesis, maturation and releasing is shown in the Figure 1 (Sohel, 2016). miRNAs can be produced through a number of non-canonical pathways, in which the protein complex of Drosha and DGCR8 isn’t required. The cleavage of the intermediate precursor can be performed by Dicer (Havens et al., 2012). However, miR-451 is processed by Drosha, but Dicer cleavage is bypassed. Instead, pre-miRNA is cleaved by the Ago catalytic center (Cheloufi et al., 2010).

### miRNA Mechanism of Action

miRNAs work as posttranscriptional gene regulators by binding to the 3′UTR, 5′-UTR regions or to the coding sequence, which mainly leads to negative expression regulation or exonucleolytic mRNA cleavage. Rarely, a positive expression regulation occurs. Alternatively, miRNAs can serve as transcription factors, when re-localized in the nucleus (Berindan-Neagoe et al., 2014). It has been determined that miRNAs can function either as oncogenes or tumor suppressors, depending on cellular context. They can block the access to mRNA, increase its degradation or induce the gene expression by binding to the complementary promoter sequence (Cheloufi et al., 2010; Havens et al., 2012; Berindan-Neagoe et al., 2014). The full-length complementary to the target mRNA isn’t required, therefore a single miRNA can manage the expression of over 100 genes (Gunasekharan and Laimins, 2013). While the vast majority of human protein-coding genes are under regulation of these small molecules, abnormal miRNA expression profoundly determines a wide variety of cellular pathways governing human malignancies, such as cell proliferation, apoptosis, metastasis, and drug response. Thus, miRNAs influence the regulation of cellular networks and have significant impact on human health (Leva et al., 2014). A number of reports have shown the linkage of aberrant miRNA expression with cancer development and changes in cancer-associated genes (Lages et al., 2012; Lan et al., 2015). However, the assessment of miRNAs expression by analyzing tissue specimens, would require invasive biopsies. In fact, miRNAs appear to be present circulating in the body fluids as a result of apoptosis or necrotic cell death. The molecules can be released encapsulated in the cell-free lipid carriers (microvesicles, exosomes, and apoptotic bodies) or bound to protein complexes, with Argonaute or high-density lipoproteins (HDL), which enables them to evade the RNase digestion and remain stable in the circulation (Schwarzenbach et al., 2014; Larrea et al., 2016). miRNA can be also actively secreted and considered to work as mediators of intercellular communication between cells (Schwarzenbach et al., 2014). The molecules are probably internalized through endocytosis by the recipient cells, where they can bind to target mRNA and fulfill their biological functions (Larrea et al., 2016). Extracellular miRNA expression is tissue-specific and shows unique patterns in different cancer tissues (Schwarzenbach et al., 2014). Thus, circulating miRNA levels may reflect the condition of tumor tissue. Expression changes in the body fluids as a result of tissue injury or disease make the molecules potentially useful biomarkers for cancer diagnosis (Schwarzenbach et al., 2014). miRNA molecules remain remarkably stable in formalin-fixed and paraffin-embedded cancer tissues and also in human plasma, increasing the probability of identifying the disease state (Schwarzenbach et al., 2014; Lan et al., 2015). Subsequently, miRNAs comprise a class of gene expression modulating molecules which may function as effective biomarkers enabling to monitor the therapy effectiveness. Over the past decade, numerous miRNA analyses demonstrated the linkage of its aberrant expression profiles, either downregulated or upregulated, with development of different cancers, including lymphoma (Di Lisio et al., 2012), breast cancer (BC) (Matamala et al., 2016), colorectal cancer (CRC) (Dong et al., 2011), and prostate cancer (Fabris et al., 2016). More recently, miRNAs have been demonstrated to be associated with anticancer drug resistance (Hayes et al., 2014).

## Materials and Methods

In this review, we summarized the latest reports concerning miRNAs as biomarkers for both diagnosis and surgical treatment monitoring. Published studies were identified through a review of literature available on PubMed. We used keywords “cancer,” “miRNA,” “treatment response,” and “biomarker” and selected research studies published from 2008 to 2018 concerning measurements of circulating miRNA expression in plasma from cancer patients treated with surgery. We searched for analyses including specifically circulating miRNA-based cancer biomarkers determination, miRNA profiling, and evaluation of molecules’ deregulations in cancer patients. In addition, the literature citation of each identified article was searched. Studies comprised of at least two miRNA expression measurements: before and after the surgery were taken into our consideration, regardless of the type or stage of cancer. Results were manually screened to subtract tissue miRNA analyses, cell cultures researches, non-surgical treatment methods and non-human studies. Only statically significant (*p*-value < 0.05) results were included in our review.

### miRNAs as Clinical Cancer Biomarkers

miRNAs have been proved to be deregulated in cancerous samples and many data are currently available on their expression as prognostic and treatment response factors. Several studies demonstrated changes of selected miRNA expression in serum samples from patients suffering from BC and samples from healthy individuals, as well as samples received before and after a breast-reductive surgery. Heneghan and colleagues, using real-time PCR based analysis, reported an increased miR-195 expression in 148 analyzed BC cancer patients in comparison to 44 controls. Moreover, circulating miR-195 and let-7a levels were shown to decrease in patients’ blood 2 weeks after breast reduction reaching the expression level observed in the healthy group (Heneghan et al., 2010). Schooneveld and colleagues demonstrated a significant (*p* < 0.05) differences in miR-215, miR-299-5p, and miR-411 expression levels between sera samples collected from 20 healthy controls and from 75 patients with BC, including 16 patients with untreated metastatic breast. The comparison showed downregulation of miRNAs expression in cancerous serum samples, though the expression values returned to normal 8 weeks after receiving surgical treatment (van Schooneveld et al., 2012). Another study performed on 170 BC patients by Enders et al. showed statistically significant (*p* < 0.005) decrease of plasma levels of the miRNAs. Expression changes were observed 14 days after the surgical removal of the tumor. According to the authors, the study provided a combined plasma biomarker of miR-451 and miR-145, which facilitates distinguishing BC patients from healthy volunteers with high efficiency (*p* < 0.0001) (Ng et al., 2013). Ng et al. revealed that overexpressed (*p* < 0.0005) miR-17-3p and miR-92a in CRC patients’ plasma decreased (*p* < 0.05) in 10 of 25 postoperative samples, 7 days after the tumor removal, compared with samples examined before the surgery. Further study indicated that miR-92 discriminates CRC patients from gastric cancer (GC) patients, ones with inflammatory bowel disease and healthy individuals, therefore the isoform is considered as a promising molecular biomarker of CRC (Ng et al., 2009). Plasma levels of miR-221 was assessed in 47 cancer patients and 30 healthy controls by real-time RT–PCR in the study of Kawaguchi et al. Isoform was reported to be upregulated in pancreatic cancer (PC) group comparing to controls, moreover its level significantly (*p* = 0.018) decreased 1 month after the pancreatectomy (Kawaguchi et al., 2013). A study of 90 patients before surgery, 50 patients 1 month after the surgery and 90 healthy controls, carried out by Lian et al. revealed four circulating miRNAs with high potential to become an osteosarcoma diagnostic panel. miR-195–5p, miR-199a-3p, miR-320a, and miR-374a-5p were shown to be significantly (*p* < 0.0001) overexpressed in the pre-operative plasma samples in comparison to the control subject, however, the expression level of these 4 isoforms dropped 1 month after the surgery (*p* < 0.0001) (Lian et al., 2015). Komatsu et al. conducted a quantitative RT–PCR analysis of oncogenic miR-21 and miR-375 in plasma samples from 50 patients suffering from esophageal squamous cell carcinoma (ESCC) and 20 healthy individuals. The study showed significantly (*p* = 0.0218) higher concentration of miR-21 and strongly (*p* = 0.0052) lower level of miR-375 in ESCC patients’ plasma in comparison to healthy volunteers. The concentration levels of plasma miR-21 significantly (*p* = 0.0058) declined in samples from patients 1 month after curative esophagectomy. However, miR-375 concentration didn’t change with statistical significance in patients who underwent the surgery (*p* = 0.2626) (Konishi et al., 2012). Another report identified a high deregulation of miR-25 expression in 105 ESCC patients in comparison to 50 control subjects and showed plasma miRNA levels were remarkably higher in ESCC patients (*p* < 0.0001). The analysis of plasma postoperative samples 1 month after undergoing esophagectomy and endoscopic resection demonstrated a significant (*p* = 0.00014) decrease of up-regulated miR-25 in cancer patients. Additionally, a remarkable (*p* = 0.0145) re-elevation of plasma miRNA level at recurrence was observed (Komatsu et al., 2014). Kanemaru and colleagues observed high (*p* < 0.0001) upregulation of circulating miR-221 expression in serum samples derived from 94 patients with malignant melanoma in comparison to 20 healthy subjects. Further, a quantitative real-time PCR analysis of eight patients, who underwent primary melanoma excision, indicated a significant (*p* < 0.001) decrease of miR-221. miRNA expression increased again in seven of the eight patients with cancer recurrence (Kanemaru et al., 2011). However, the study lacks of information what period after the operation the second plasma collection was performed. Another study revealed potential miRNAs biomarkers for lung carcinoma patients. Han-Bo Le and colleagues compared the sera expression levels of miR-21 and miR-24 in 50 normal controls and 82 patients before and 10 days after the surgery. Investigated miRNAs were selected as serum biomarkers, according to a significant (*p* = 0.0004 and *p* < 0.0001, respectively) expression decrease in postoperative samples (Le et al., 2012). A report concerning four isoforms of miRNA in non-small cell lung cancer (NSCLC) plasma patients demonstrated that miR-9, miR-16, miR-205, and miR-486 may serve as NSCLC biomarkers. miRNAs levels were assessed before treatment in 61 patients, follow up measurement was performed 1 month postoperatively in 37 patients and 1 year after surgery in 14 patients. Cancer group showed significantly increased plasma levels of miR-16 and miR-486 comparing to the control group. Higher expression of miR-205 was found only in squamous cell carcinoma patients and miR-9 wasn’t found deregulated. Following tumor resection, miR-16, miR-9, and miR-205 levels decreased, whereas miR-486 expression appeared to be elevated (Sromek et al., 2017). However, parallel study of Wanshuai et al. contradicted these outcomes. Although miR-486 was shown to distinguish NSCLC cancer patients from healthy controls, its expression was significantly increased in cancer cohort and dropped 7–10 days postoperatively (Li et al., 2015). Konishi and colleagues found potential GC biomarkers based on plasma miR-451 and miR-486 expression levels. The expression of miRNAs was shown to be up-regulated in cancerous samples in comparison to healthy controls. Further qRT–PCR analysis showed a significant decrease of miRNAs expression in patients 1–2 months after the operation, comparing with levels observed in sera before the surgery (Konishi et al., 2012). In a more recent study of Chen et al. plasma expression of miR-204 was compared between 115 GC and 40 healthy individuals. Level of circulating miR-204 was significantly lower (*p* < 0.01) in the cancer group and increased (*p* < 0.01) after receiving the tumor resection (Chen et al., 2016). However, the study lacks of information what period after the operation the second plasma collection was performed. Investigating of serum samples obtained from 82 renal cell carcinoma (RCC) patients and 80 healthy individuals, Zhang et al. captured abnormally expressed miRNA isoforms: miR-210 and miR-1233. Both miR-210 and miR-1233 expression levels were significantly (*p* < 0.001 both) elevated in cancer patients in comparison to healthy controls. Seven days after surgical tumor removal, the expression levels of miRNA isoforms in sera were significantly lower than in the preoperative samples (*p* = 0.004 and *p* = 0.008, respectively) (Zhang et al., 2018). Both miR-378 and miR-210 were reported to be significantly elevated in sera of RCC patients differing them from the healthy donors. Fedorko et al. (2015) observed a marked decrease (*p* < 0.0001 both) of miRNAs in patients cohort 3 months after a radical nephrectomy. Similar observation was made by Lou and colleagues, who examined 106 RCC plasma samples and found an up-regulation of miR-144-3p isoform in comparison to 123 healthy individuals. In addition, a marked (*p* < 0.0001 both) decrease of miR144-3p levels 7 days after nephrectomy in all patients’ blood was noted (Lou et al., 2017). MiR-21 and miR-106a are expected to be another renal cancer biomarkers. Serum levels of the molecules were increased (*p* = 0.001) in 30 RCC blood samples comparing to the control group. 1 month after surgery expression of examined molecules was significantly reduced (*p* = 0.032 and *p* < 0.001, respectively) (Tusong et al., 2017). Important differences between serum levels of miR-222, miR-221, and miR-146b in samples derived from 85 patients newly diagnosed with papillary thyroid carcinoma (PTC), 35 cases of benign thyroid nodules (BTN) and 40 control subjects were reported. Researchers observed a high overexpression of miRNAs in PTC samples, additionally similar miR-221 up-regulation was identified in the serum samples from BTN patients (*p* < 0.001 and *p* < 0.001, respectively). Authors monitored expression level changes of miR-222, miR-221, and miR-146b 1, 3, 6, and 12 months postoperatively in both groups by RT-qPCR analysis. Levels of overexpressed miR-222, miR-221, and miR-146b plummeted 1 month following the surgery (hemithyroidectomy or total thyroidectomy) comparing to the preoperative level in PTC patients (*p* < 0.01). All 3 miRNAs progressively decreased between 3 and 6 months after the surgery in the PTC group (*p* < 0.05). Renewed increase of investigated miRNAs expression levels correlated with recurrence, comparing to healthy controls and the recurrence-free survivors (*p* < 0.001 and *p* < 0.001, respectively) (Zhang et al., 2017). Four oncogenic miRNAs, miR-151, miR155, miR-191, and miR-224, were confirmed to be overexpressed in hepatocellular cancer patients’ plasma. A small scale analysis conducted on 20 hepatocellular cancer patients and 20 healthy controls identified a significant upregulation of miRNA isoforms (*p* = 0.0186, *p* = 0.0206, *p* = 0.0118, and *p* < 0.0001, respectively). Further investigation of postoperative miRNAs expression showed a significant reduction of plasma miR-224 (*p* = 0.0058) 1 month after the curative hepatectomy (Okajima et al., 2016). Yamamoto et al. (2009) revealed a similar pattern investigating serum miR-500, which appeared to be remarkably overexpressed (*p* < 0.001) in 40 hepatocellular carcinoma patients comparing to matching non-cancerous samples, and decreased subsequently 6 months after the surgical treatment. Most commonly deregulated miRNAs, which expression changed due to surgical anticancer treatment are summarized in Table 1.

**Table 1:** The list of circulating miRNAs isoforms most commonly associated with cancer diseases and postoperative effect on their expression.

miRNA isoform	Cancer	Postoperative effect on miRNA expression
miR-221	Breast cancer (BC), melanoma, thyroid, renal cell carcinoma	Decrease
miR-375	BC, pancreatic, esophageal	Decrease
miR-21	Esophageal, lung cancer	Decrease
miR-486	Non-small cell lung cancer	Increase or decrease
	Gastric cancer	Decrease


## Discussion and Conclusion

Research on miRNAs as cancer biomarkers is relatively new, yet, promising. Our review presented 21 articles concerning 10 cancer diseases and 31 different miRNAs pointed out as potential anticancer treatment response biomarkers.

For the variety of cancers resistant to conventional chemotherapy and radiation therapy, surgery remains the only applicable curative treatment. Up to 50% of CRC patients (Mirnezami et al., 2011) and from 30 to 55% of NSCLC patients (Uramoto and Tanaka, 2014) may develop recurrence after the tumor resection. Effective biomarkers for non-invasive, early detection would contribute to the reduction of cancer mortality. A number of studies demonstrated that miRNAs circulate in blood and can be easily obtained, and measured through well known, low cost methods like RT-PCR. Molecules are detectable even in a small sample amount (Cappelletti et al., 2015). Stability in body fluids, easy obtainment and detection are important for their future clinical applications (Schwarzenbach et al., 2014; Lan et al., 2015). Any deregulation in miRNA expression may result in cancer development. All the data suggests a putative role for miRNAs in cell communication during pathological events, therefore blood circulating miRNAs can be useful for diagnosis, prognosis, and monitoring the efficacy of treatment. Genome-wide screening can help identifying novel cancer-associated miRNAs, which could serve as diagnostic markers or cancer recurrence indicators. However, several studies highlighted some important issues, which future studies have to take into consideration. A results discrepancy among studies is observed. There are only few correlative reports with clinical outcome, which can be associated with lack of common standard procedure and normalization. Moreover, it was demonstrated that the choice of substrate for isolation is crucial for credible results (Singh et al., 2016). miRNA concentrations in serum and plasma obtained from the same patient may vary leading to different miRNA profiles. The relation between tissue expression and cell-free circulating miRNA isn’t clear, too. It is speculated if the extracellular level is a result of expression perturbations in the tumor cell, or a non-specific consequence of the neoplasm occurrence or other environment factors (Witwer, 2015). Regarding reviewed studies, postoperative miRNA-based follow-up information may be disrupted by miRNA revealed to the extracellular space in the consequence of tissue injury or inflammation caused by surgical intervention. A report on rat models of Laterza et al. (2009) demonstrated that postoperative plasma miRNA levels corresponded to injuries in liver, muscles, and brain tissues. This factor should be taken into consideration during further studies. It is critical to extend current understanding of miRNA biology and determine the exact mechanisms in which miRNAs execute specific functions. Despite several limitations, miRNAs are promising molecules for clinical biomarkers, as demonstrated by the burst of scientific reports on miRNA-based “liquid biopsies.” All the findings require deeper verification due to poor results overlap among the reports. Additionally, a standardized procedure for sample choice, preparation and processing is necessary to avoid false results. In conclusion, the studies mentioned above highlight the potential value of miRNAs in cancer diagnosis and monitoring patients’ response to the therapy, however, further large-scale evaluation is necessary. A number of analyses on miRNA expression before and after the surgical therapy is insufficient to confront the data and evaluate their credibility and applicability. However, these data revealed a huge potential of miRNA molecules to become prevalent, non-invasive and effective biomarkers. There is also a fairly consistent evidence that miRNAs can work as biomarkers giving the clinical follow up information on the efficiency of curative anticancer therapy. However, in the future, there should be more observational studies published concerning similar miRNAs expression analyses, so a meta-analysis may become possible.

## Author Contributions

Both authors contributed to the design and writing of the manuscript.

## Conflict of Interest Statement

The authors declare that the research was conducted in the absence of any commercial or financial relationships that could be construed as a potential conflict of interest.

## References

[B1] Berindan-Neagoe I. Monroig P. Pasculli B. Calin G. A. (2014). MicroRNAome genome: a treasure for cancer diagnosis and therapy. *CA Cancer J. Clin.* 64 311–336. 10.3322/caac.21244 25104502 PMC4461198

[B2] Cappelletti V. Appierto V. Tiberio P. Fina E. Callari M. Daidone M. G. (2015). Circulating biomarkers for prediction of treatment response. *J. Natl. Cancer Inst. Monogr.* 2015 60–63. 10.1093/jncimonographs/lgv006 26063889

[B3] Cheloufi S. Dos Santos C. O. Chong M. M. W. Hannon G. J. (2010). A dicer-independent miRNA biogenesis pathway that requires Ago catalysis. *Nature* 465 584–589. 10.1038/nature09092 20424607 PMC2995450

[B4] Chen X. Liu X. S. Liu H. Y. Lu Y. Y. Li Y. (2016). Reduced expression of serum miR-204 predicts poor prognosis of gastric cancer. *Genet. Mol. Res.* 15:5027702. 10.4238/gmr.15027702 27173244

[B5] Di Lisio L. Sánchez-Beato M. Gómez-López G. Rodríguez M. E. Montes-Moreno S. Mollejo M. (2012). MicroRNA signatures in B-cell lymphomas. *Blood Cancer J.* 2 1–9. 10.1038/bcj.2012.1 22829247 PMC3288280

[B6] Dong Y. Wu W. K. K. Wu C. W. Sung J. J. Y. Yu J. Ng S. S. M. (2011). MicroRNA dysregulation in colorectal cancer: a clinical perspective. *Br. J. Cancer* 104 893–898. 10.1038/bjc.2011.57 21364594 PMC3065287

[B7] Fabris L. Ceder Y. Chinnaiyan A. M. Jenster G. W. Sorensen K. D. Tomlins S. (2016). The potential of MicroRNAs as prostate cancer biomarkers. *Eur. Urol.* 70 312–322. 10.1016/j.eururo.2015.12.054 26806656 PMC4927364

[B8] Fedorko M. Stanik M. Iliev R. Redova-Lojova M. Machackova T. Svoboda M. (2015). Combination of MiR-378 and MiR-210 serum levels enables sensitive detection of renal cell carcinoma. *Int. J. Mol. Sci.* 16 23382–23389. 10.3390/ijms161023382 26426010 PMC4632704

[B9] Gunasekharan V. Laimins L. A. (2013). Human papillomaviruses modulate MicroRNA 145 expression to directly control genome amplification. *J. Virol.* 87 6037–6043. 10.1128/JVI.00153-13 23468503 PMC3648148

[B10] Havens M. A. Reich A. A. Duelli D. M. Hastings M. L. (2012). Biogenesis of mammalian microRNAs by a non-canonical processing pathway. *Nucleic Acids Res.* 40 4626–4640. 10.1093/nar/gks026 22270084 PMC3378869

[B11] Hayes J. Peruzzi P. P. Lawler S. (2014). MicroRNAs in cancer: biomarkers, functions and therapy. *Trends Mol. Med.* 20 460–469. 10.1016/j.molmed.2014.06.005 25027972

[B12] Heneghan H. M. Miller N. Lowery A. J. Sweeney K. J. Newell J. Kerin M. J. (2010). Circulating micrornas as novel minimally invasive biomarkers for breast cancer. *Ann. Surg.* 251 499–505. 10.1097/SLA.0b013e3181cc939f 20134314

[B13] Heywood S. M. Kennedy D. S. Bester A. J. (1974). Separation of specific initiation factors involved in the translation of myosin and myoglobin messenger RNAs and the isolation of a new RNA involved in translation. *Proc. Natl. Acad. Sci. U.S.A.* 71 2428–2431. 10.1073/pnas.71.6.2428 4526305 PMC388470

[B14] Kanemaru H. Fukushima S. Yamashita J. Honda N. Oyama R. Kakimoto A. (2011). The circulating microRNA-221 level in patients with malignant melanoma as a new tumor marker. *J. Dermatol. Sci.* 61 187–193. 10.1016/j.jdermsci.2010.12.010 21273047

[B15] Kawaguchi T. Komatsu S. Ichikawa D. Morimura R. Tsujiura M. Konishi H. (2013). Clinical impact of circulating miR-221 in plasma of patients with pancreatic cancer. *Br. J. Cancer* 108 361–369. 10.1038/bjc.2012.546 23329235 PMC3566805

[B16] Komatsu S. Ichikawa D. Hirajima S. Kawaguchi T. Miyamae M. Okajima W. (2014). Plasma microRNA profiles: identification of miR-25 as a novel diagnostic and monitoring biomarker in oesophageal squamous cell carcinoma. *Br. J. Cancer* 111 1614–1624. 10.1038/bjc.2014.451 25117812 PMC4200091

[B17] Konishi H. Ichikawa D. Komatsu S. Shiozaki A. Tsujiura M. Takeshita H. (2012). Detection of gastric cancer-associated microRNAs on microRNA microarray comparing pre- and post-operative plasma. *Br. J. Cancer* 106 740–747. 10.1038/bjc.2011.588 22262318 PMC3322946

[B18] Lages E. Guttin A. Nesr H. Lages E. Guttin A. Nesr H. (2012). MicroRNAs? molecular features and role in cancer. *Front. Biosci.* 17 2508–2540. 10.2741/4068 PMC3815439 22652795

[B19] Lan H. Lu H. Wang X. Jin H. (2015). MicroRNAs as potential biomarkers in cancer: opportunities and challenges. *Biomed. Res. Int.* 2015:125094. 10.1155/2015/125094 25874201 PMC4385606

[B20] Larrea E. Sole C. Manterola L. Goicoechea I. Armesto M. Arestin M. (2016). New concepts in cancer biomarkers: circulating miRNAs in liquid biopsies. *Int. J. Mol. Sci.* 17:E627. 10.3390/ijms17050627 27128908 PMC4881453

[B21] Laterza O. F. Lim L. Garrett-Engele P. W. Vlasakova K. Muniappa N. Tanaka W. K. (2009). Plasma microRNAs as sensitive and specific biomarkers of tissue injury. *Clin. Chem.* 55 1977–1983. 10.1373/clinchem.2009.131797 19745058

[B22] Le H. B. Zhu W. Y. Chen D. D. He J. Y. Huang Y. Y. Liu X. G. (2012). Evaluation of dynamic change of serum miR-21 and miR-24 in pre- and post-operative lung carcinoma patients. *Med. Oncol.* 29 3190–3197. 10.1007/s12032-012-0303-z 22782668

[B23] Leva G. Di Garofalo M. Croce C. M. (2014). microRNAs in cancer. *Annu. Rev. Pathol.* 9 287–314. 10.1146/annurev-pathol-012513-104715 24079833 PMC4009396

[B24] Li W. Wang Y. Zhang Q. Tang L. Liu X. Dai Y. (2015). MicroRNA-486 as a biomarker for early diagnosis and recurrence of non-small cell lung cancer. *PLoS One* 10:e0134220. 10.1371/journal.pone.0134220 26237047 PMC4523212

[B25] Lian F. Cui Y. Zhou C. Gao K. Wu L. (2015). Identification of a plasma four-microRNA panel as potential noninvasive biomarker for osteosarcoma. *PLoS One* 10:e0121499. 10.1371/journal.pone.0121499 25775010 PMC4361617

[B26] Lou N. Ruan A. M. Qiu B. Bao L. Xu Y. C. Zhao Y. (2017). miR-144-3p as a novel plasma diagnostic biomarker for clear cell renal cell carcinoma. *Urol. Oncol.* 35 36.e7–36.e14. 10.1016/j.urolonc.2016.07.012 27633984

[B27] Matamala N. Teresa Vargas M. González-Cámpora R. Ignacio Arias J. Menéndez P. Andrés-León E. (2016). MicroRNA deregulation in triple negative breast cancer reveals a role of miR-498 in regulating BRCA1 expression. *Oncotarget* 7 20068–20079. 10.18632/oncotarget.7705 26933805 PMC4991439

[B28] Mirnezami A. Mirnezami R. Chandrakumaran K. Sasapu K. Sagar P. Finan P. (2011). Increased local recurrence and reduced survival from colorectal cancer following anastomotic leak: systematic review and meta-analysis. *Ann. Surg.* 253 890–899. 10.1097/SLA.0b013e3182128929 21394013

[B29] Ng E. K. O. Chong W. W. S. Jin H. Lam E. K. Y. Shin V. Y. Yu J. (2009). Differential expression of microRNAs in plasma of patients with colorectal cancer: a potential marker for colorectal cancer screening. *Gut* 58 1375–1381. 10.1136/gut.2008.167817 19201770

[B30] Ng E. K. O. Li R. Shin V. Y. Jin H. C. Leung C. P. H. Ma E. S. K. (2013). Circulating microRNAs as specific biomarkers for breast cancer detection. *PLoS One* 8:e53141. 10.1371/journal.pone.0053141 23301032 PMC3536802

[B31] Okajima W. Komatsu S. Ichikawa D. Miyamae M. Kawaguchi T. Hirajima S. (2016). Circulating microRNA profiles in plasma: Identification of miR-224 as a novel diagnostic biomarker in hepatocellular carcinoma independent of hepatic function. *Oncotarget* 7 53820–53836. 10.18632/oncotarget.10781 27462777 PMC5288224

[B32] Schwarzenbach H. Nishida N. Calin G. A. Pantel K. (2014). Clinical relevance of circulating cell-free microRNAs in cancer. *Nat. Rev. Clin. Oncol.* 11 145–156. 10.1038/nrclinonc.2014.5 24492836

[B33] Singh R. Ramasubramanian B. Kanji S. Chakraborty A. R. Haque S. J. Chakravarti A. (2016). Circulating microRNAs in cancer: hope or hype? *Cancer Lett.* 381 113–121. 10.1016/j.canlet.2016.07.002 27471105

[B34] Sohel M. H. (2016). Extracellular/circulating MicroRNAs: release mechanisms, functions and challenges. *Achiev. Life Sci.* 10 175–186. 10.1016/j.als.2016.11.007

[B35] Sromek M. Glogowski M. Chechlinska M. Kulinczak M. Szafron L. Zakrzewska K. (2017). Changes in plasma miR-9, miR-16, miR-205 and miR-486 levels after non-small cell lung cancer resection. *Cell. Oncol.* 40 529–536. 10.1007/s13402-017-0334-8 28634901 PMC13001572

[B36] Tusong H. Maolakuerban N. Guan J. Rexiati M. Wang W. G. Azhati B. (2017). Functional analysis of serum microRNAs miR-21 and miR-106a in renal cell carcinoma. *Cancer Biomark.* 18 79–85. 10.3233/CBM-160676 27814278 PMC13020616

[B37] Uramoto H. Tanaka F. (2014). Recurrence after surgery in patients with NSCLC. *Transl. Lung. Cancer Res.* 3 242–249. 10.3978/j.issn.2218-6751.2013.12.05 25806307 PMC4367696

[B38] van Rooij E. Kauppinen S. (2014). Development of microRNA therapeutics is coming of age. *EMBO Mol. Med.* 6 851–864. 10.15252/emmm.201100899 24935956 PMC4119351

[B39] van Schooneveld E. Wouters M. C. A. Van der Auwera I. Peeters D. J. Wildiers H. Van Dam P. A. (2012). Expression profiling of cancerous and normal breast tissues identifies microRNAs that are differentially expressed in serum from patients with (metastatic) breast cancer and healthy volunteers. *Breast Cancer Res.* 14:R34. 10.1186/bcr3127 22353773 PMC3496152

[B40] Witwer K. W. (2015). Circulating MicroRNA biomarker studies: pitfalls and potential solutions. *Clin. Chem.* 61 56–63. 10.1373/clinchem.2014.221341 25391989

[B41] Yamamoto Y. Kosaka N. Tanaka M. Koizumi F. Kanai Y. Mizutani T. (2009). MicroRNA-500 as a potential diagnostic marker for hepatocellular carcinoma. *Biomarkers* 14 529–538. 10.3109/13547500903150771 19863192

[B42] Zhang W. Ni M. Su Y. Wang H. Zhu S. Zhao A. (2018). MicroRNAs in serum exosomes as potential biomarkers in clear-cell renal cell carcinoma. *Eur. Urol. Focus* 4 412–419. 10.1016/j.euf.2016.09.007 28753793

[B43] Zhang Y. Xu D. Pan J. Yang Z. Chen M. Han J. (2017). Dynamic monitoring of circulating micrornas as a predictive biomarker for the diagnosis and recurrence of papillary thyroid carcinoma. *Oncol. Lett.* 13 4252–4266. 10.3892/ol.2017.6028 28599426 PMC5452941

